# Phenazine 5,10-dioxide analogues as potential therapeutics in AML: Efficacy on patient-derived blasts, in zebrafish larvae xenografts and synergy with venetoclax

**DOI:** 10.1016/j.tranon.2025.102628

**Published:** 2025-12-06

**Authors:** Ingeborg Nerbø Reiten, Reidun Aesoy, Jan-Lukas Førde, Goraksha Machhindra Khose, Elvar Örn Viktorsson, Øystein Bruserud, Pål Rongved, Håkon Reikvam, Lars Herfindal

**Affiliations:** a Centre for Pharmacy, Department of Clinical Science, University of Bergen, Norway; b School of Health Sciences, Faculty of Pharmaceutical Sciences, University of Iceland, Iceland; c School of Pharmacy, Department of Pharmaceutical Chemistry, University of Oslo, Norway; d Department of Medicine Haukeland University Hospital, Bergen, Norway; e K.G. Jebsen Center for Myeloid Malignancies, Department of Clinical Science, University of Bergen, Bergen, Norway

**Keywords:** Acute myeloid leukemia, Phenazines, Venetoclax,, Patient blasts, Zebrafish larvae

## Abstract

Although several new therapies against acute myeloid leukaemia (AML) have emerged the past years, patients who are ineligible for intensive chemotherapy are still treated with less effective treatments to minimise therapy-associated mortality. Several phenazine 5,10-dioxide derivates have previously demonstrated to selectively induce apoptosis in human AML cells. In the present work, we have continued investigations on phenazine 5,10-dioxides to reveal their therapeutic potential in AML using in vitro and in vivo experiments. From a panel of primary AML blasts from 61 non-selected patients, 58 showed high or intermediate response to treatment with the phenazine 5,10-dioxides. This included blasts with biological characteristics associated with poor prognosis, such as *FLT3* internal tandem duplication, *NPM-1* wild type, CD34^+^, and adverse cytogenetics. The phenazine 5,10-dioxides cytotoxicity towards primary blasts correlated with the blast’s sensitivity to daunorubicin, presumably due to similar mode of action towards AML cells. Three phenazine 5,10-dioxides had low toxicity in zebrafish larvae, and from these, two were found effective towards zebrafish larvae AML xenografts. Additionally, synergism with the AML drug venetoclax (VTX) was found in the AML cell lines MOLM-13 and MV4–11. The efficacy of phenazine 5,10-dioxides towards primary AML blasts, synergism with VTX and low toxicity in effective concentrations in zebrafish larva AML xenografts suggests potential for these compounds in future AML therapy for patients unfit for intensive chemotherapy.

## Introduction

Phenazines are aromatic *N*-heterocyclic compounds first isolated in the mid-19th century from the bacterium *Pseudomonas aeruginosa* [1]. In nature, they are mainly found in *Pseudomonas* and *Streptomyces* species [2], but since their discovery, >6000 synthesised phenazines have been described, and many investigated for both antimicrobial and anti-cancer properties [3]. The natural phenazine 5,10-dioxide compound iodinin (1,6-dihydroxyphenazine 5,10-dioxide) is named for its deep purple colour and was first isolated in 1939 [4]. Iodinin selectively induces apoptosis in several acute myeloid leukaemia (AML) cancer cell lines and patient derived blasts [5]. It is, however, inadequate as a drug molecule due to poor aqueous solubility caused by two intramolecular ion-dipole bonds. To improve the properties of the molecule, Viktorsson and coworkers synthesised a panel of phenazine 5,10-dioxide analogues [6,7]. Several of the new compounds showed improved physiochemical properties like enhanced solubility and membrane permeability compared to iodinin. In addition, some compounds had improved cytotoxic potential towards the AML cell line MOLM-13, while maintaining selectivity relative to non-malignant cell lines [[6], [8]]. The phenazine 5,10-dioxide analogues thus represent promising new modalities for AML therapy, for instance to treat patients who have low tolerance to standard AML therapy.

The 7 + 3 intensive induction treatment in AML, which consists of 7 days of cytarabine and 3 days of an anthracycline, has remained essentially the same since its introduction >50 years ago, and is still the preferred induction treatment in AML [9,10]. However, some new treatment modalities and therapeutics have emerged in the past few years. For instance, the FLAG-IDA regimen consisting of fludarabine, cytarabine, granulocyte-colony stimulating factors (G-CSF) and idarubicin can be used as an alternative intensive induction therapy [10]. Additionally, the *FLT3*-inhibitor midostaurin is now recommended alongside induction therapy for patients with *FLT3* mutated AML and has improved outcomes for these patients. In 2018, the BCL-2 inhibitor venetoclax (VTX) was granted an accelerated FDA-approval in combination with hypomethylating agents (HMA) or low-dose cytarabine in the treatment of AML for patient’s ineligible for intensive chemotherapy [11]. The approval came after two phase Ib/II studies demonstrated increased complete remission rates and overall survival compared to HMA and LDAC alone [12,13]. The combination of VTX and HMA (azacitidine) increased the median overall survival to 14.7 months compared to 9.6 months for azacitidine alone [14] and VTX-based regimen has since become the new standard of treatment for these patients [11]. Even so, this therapy regimen is rarely curative and is likely to increase the risk of serious adverse events in the patients including febrile neutropenia and tumour lysis syndrome [15,16]. Presently, VTX continues to be investigated in combination with other AML drugs, for instance alongside the 7 + 3 therapy or the FLAG-IDA regimen with promising results, reviewed in [17]. The last few years, new drugs like gilteritinib and quizartinib has emerged as useful in consolidation or maintenance therapy, often in combination with traditional chemotherapy like cytarabine or an anthracycline (see references [18] and [19] for recent reviews). These abovementioned new agents are likely to become part of standard therapy for hard-to-treat AML as more clinical data are obtained.

We have previously identified structure-activity relationships between the chemical structure of various phenazine 5,10-dioxides and their activity towards AML and non-cancerous cell lines [6,[7], [8]]). The purpose of the present study was to provide further documentation of chosen AML-selective phenazine 5,10-dioxides [6,7]. In this study we focus on their cytotoxic efficacy towards primary AML blasts, and their cytotoxicity in combination with other AML drugs. Finally, we tested selected analogues for toxicity and efficacy in vivo using zebrafish larvae as a model organism. Zebrafish larvae have become an increasingly popular model system for drug discovery over the past decades. Their small size, larval transparency and rapid development in combination with similar genome and biology to humans provides a disease model that has shown to be relevant in drug development for cancers [20].

## Methods

### Chemicals and reagents

Formaldehyde (F1635), phosphate buffered saline (PBS, P4417), ammonium sulphate (A4418), WST-1 (CELLPRO-RO), ethyl 3-aminobenzoate methane-sulfonate (MS-222, E10521), Minimum Essentials Medium Eagle (M2279), Iscove’s Modified Dulbecco’s Medium (I3390), RPMI-1640 medium (R5886), penicillin-streptomycin (P0781), l-glutamine solution (G7513), foetal bovine serum (F7524) and bisbenzimide H-33,342 trihydrochloride (Hoechst 33,342, 14,533) were all from Merck Life Sciences (Darmstadt, Germany). Daunorubicin (DNR) was from Sanovi-Aventis (Paris, France). StemSpan™ SFEM medium was from Stemcell Technologies (Vancouver, Canada). The cytokines Human SCF (PeproTech #300–07), Human Flt-3 ligand (PeproTech #300–19), and Human G-CSF (PeproTech #300–23) were from Thermo Scientifics/Peprotech (Waltham, MA, USA). Lymphoprep was from Axis-Shield (Dundee, UK). Pacific Blue-AnnexinV (640,918) and propidium iodide (PI, 421,301) was from BioLegend (San Diego, CA, USA). CellTracker™ Deep Red (C34565) was from Thermo Scientific (Waltham, MA, USA).

The phenazine 5,10-dioxides used in the project (Table 1) were synthesised as described in [6,7,21].

**Table 1:** The phenazine 5,10-dioxides used in the present study. The synthesis of the compounds is described in **a**: reference [6], **b**: reference [7], and **c**: reference [21].

Compound	R_1_	R_2_	R_6_	R_7_	R_8_	Phenazine 5,10-dioxides
Iodinin (**1**)**^a^**	-OH	-H	-OH	-H	-H	
Myxin (**2**)**^a^**	-OH	-H	-OMe	-H	-H	
**3^b^**		-H	-OH	-H	-H	
**4^a^**		-H	-OH	-H	-H	
**5^a^**		-H	-OH	-H	-H	
**6^a^**		-H	-H	-Cl	-Cl	
**7^a^**		-H	-OMe	-H	-H	
**8^c^**		-Br	-H	-H	-H	
**9^a^**		-H	-H	-Me	-Me	
**10^c^**	-OMe	-H	-H	-H	-H	
**11^c^**		-I	-H	-H	-H	

### Cell culture maintenance and experimental conditions

#### AML patient derived blasts and cell death analyses

The collection and biobanking of patient cells were approved by the regional research ethics committee and conducted in accordance with the Declaration of Helsinki. Health Region III, Bergen, Norway (REK III 060.02, 215.03, 231.06 and 2015/1410 for biobanking, and REK Vest 2015/1759, 2017/305 and REK Nord 2022/480,847 for experimental use of blasts). Samples were collected after written informed consent and stored under liquid nitrogen in biobanks approved by the Norwegian Royal Ministry of Health and the Norwegian Directorate for Health and Social Affairs.

Blasts from 61 non-selected patients were used in this study. Supplementary Table 1 presents the patient information, classification, and genetic aberration of the blasts. Peripheral blood mononuclear cells (PBMC) from healthy volunteers were obtained from freshly drawn peripheral blood from the Department of Immunology and Transfusion Medicine, Haukeland University Hospital, Bergen, Norway. AML blasts and PBMCs were isolated by density gradient separation of peripheral blood. The patient samples contained at least 95 % leukemic blasts after isolation. Bone marrow (BM) aspirate from a healthy donor was kindly provided by Professor BT Gjertsen, Dept. Clinical Science, University of Bergen (REK Vest 2012/2247). The patient blasts were cultured in StemSpan medium supplemented with SCF, Flt-3 ligand, and G-CSF, each at a final concentration of 20 ng/mL [22,23], treated with phenazine 5,10-dioxides or daunorubicin (DNR) for 24 h before cell death was assessed by flow-cytometric analyses as described previously [24]. Viable cells were defined as Annexin V/propidium iodide double negative cells [25].

#### Culturing and maintenance of AML cell lines

Three commercially available AML cell lines were used in the study: MOLM-13, established from a 20-year-old male patient with previous myelodysplastic neoplasm (MDS) and secondary acute monocytic leukaemia (DSMZ: ACC-554) [26], OCI-AML3 (DSMZ: ACC-582), an acute myelomonocytic leukaemia cell line from a 57-year-old male patient, and MV4–11 (DSMZ: ACC-102) derived from a 10-year-old male patient with acute monocytic leukaemia. MOLM-13 and OCI-AML3 were cultured in RPMI-1640 medium while MV4–11 cells were cultured in Iscove’s Modified Dulbecco’s medium. Both media were enriched with 10 % FBS, 100 IU/mL penicillin and 0.1 mg/mL streptomycin. All three cell lines were cultured at densities between 100,000 and 1000,000 cells/mL and cultured in a humidified incubator at 37 °C and 5 % CO_2_.

#### Assessment of cytotoxic effect by metabolic activity and microscopy

To study the combination effect of phenazine 5,10-dioxides and VTX in AML cell lines, the cells were seeded in 96-well plates at 35,000 cells per well for 24-hour experiments or 20,000 cells per well for 48-hour experiments. Phenazine 5,10-dioxides and VTX, alone or in combination, were added in desired concentrations and cell plates incubated for 24 and 48 h. Thereafter, WST-1 was added to each well according to the manufacturer’s instructions and incubated for two hours. Absorbance was measured at 450 nm with 620 nm reference wavelength in a Wallac EnVision® 2130 multilabel plate reader, and this value was used to calculate relative metabolic activity compared to untreated cells. The cells were next fixed in 2 % formaldehyde with 0.01 mg/ml Hoechst 33,342 dye dissolved in PBS. Nuclear morphology was studied using a Nikon Diaphot 300 microscope to confirm that metabolic activity correlated with the presence of apoptotic nuclei (Supplementary Figure 1).

### Zebrafish larvae maintenance and experimental conditions

#### Zebrafish larvae maintenance

Zebrafish embryos of the Casper [27] and AB (ZFIN ID: ZDB-GENO-960809-7) strain were collected at the Zebrafish Facility at the Department of Biological Sciences, University of Bergen. The facility is run according to the European Convention for the Protection of Vertebrate Animals used for Experimental and Other Scientific Purposes. Embryos and larvae were kept in petri dishes containing embryo medium (4.5 mM NaCl, 0.15 mM KCl, 0.30 mM CaCl_2_ × 2H_2_O, 0.30 mM MgSO_4_ in ddH_2_O and 10 µM methyl blue) at 28 °C in a humidified incubator. The petri dishes were checked daily for dead embryos or debris. At two days post fertilization (dpf) at the long pec stage [28], unhatched zebrafish larvae were manually dechorionated using fine forceps. Within 120 h post fertilization (hpf) the zebrafish larvae were euthanized by placing the petri dishes on ice for 20 min before being transferred to −20 °C overnight.

#### Determination of toxicity in zebrafish larvae

Phenazine 5,10-dioxides were dissolved in DMSO to give final concentrations between 5 and 20 mM and administered to the zebrafish larvae by diluting the stock solutions in the embryo medium. All compounds were tested up to a maximal concentration of 100 µM.

To evaluate the toxicity of VTX, the drug was dissolved in DMSO to 1 mM and diluted to 100 µM in PBS. Due to the high molecular weight and low permeability of VTX [29], the drug was administered via intravenous injections into the posterior cardinal vein. The control group received an intravenous injection of 10 % DMSO diluted in PBS, which was well tolerated by the larvae.

Zebrafish larvae heart rate was analysed as described by Førde et al. [30]. To examine zebrafish larvae for morphological abnormalities, they were imaged using a Nikon Diaphot 300 inverted microscope fitted with a Nikon DS-Fi3 microscope camera and DS-L4 camera control unit.

#### Transplantation of MOLM-13 cells into zebrafish larvae

Prior to transplantation into zebrafish larvae, MOLM-13 cells were stained with the fluorescent marker CellTracker™ Deep Red. The cell suspension was centrifuged at 300 relative centrifugal force (RCF) for 5 min, and the medium removed. Thereafter, the cells were resuspended in serum-free RPMI-1640 medium containing 1.5 µM CellTracker™ Deep Red and incubated for 30 min at 37 °C. Excess dye was removed by centrifugation for five minutes at 300 RCF, removal of the medium and the cells were resuspended in fresh RPMI-medium to a final concentration of 10 million cells/mL. Intravenous injection of MOLM-13 cells into zebrafish larvae was performed as described in Førde et al. [30]. The zebrafish larvae xenografts were incubated at 31.5 °C.

#### Quantification of tumour burden in zebrafish larvae

For quantification of tumour burden in zebrafish larvae a Nikon Ti-E inverted microscope fitted with an Andor Dragonfly 505 spinning disk confocal unit and iXon 888 Life EMCCD camera setup was used. The imaging was performed at 10x magnification and 4 µm intervals in the z-direction. Prior to imaging, the zebrafish larvae were anesthetized in 0.7 mM MS-222 and placed in chambered coverslips. The zebrafish larvae were scored based on the number of cells successfully injected and divided into treatment or control groups. Zebrafish larvae with <10 injected cancer cells were excluded from experiments.

The Fiji macro described in [30] was used to determine MOLM-13 cancer cell burden in zebrafish larvae. In brief, this macro collects and flattens a confocal image stack. After defining the boundary of the larva and segmentation from the background, the cells are segmented so that individual cells can be quantified (both size and number). Both total volume and cell count of the MOLM-13 xenograft was used to determine efficacy of treatment. The macro source code is available for download at https://zenodo.org/records/7383160 and the source code at https://doi.org/10.5281/zenodo.7383160.

### Statistical analyses

Statistical analyses were performed using IBM SPSS for Windows Ver. 29. One-way ANOVA with Tukey’s honest significant difference post-hoc test was used to determine differences in zebrafish larvae heart rate and cell metabolic activity. Two-sample two-sided Student’s *t*-test was used to evaluate treatment response in zebrafish larvae xenografts. Welch’s *t*-test was performed for groups with unequal variance estimated by Levene’s test. Two-way ANOVA was used to evaluate interaction between the effect of phenazine 5,10-dioxides and VTX in zebrafish larvae xenografts. Correlation between WST-1 and microscopical data, and patient blasts response to treatment was analysed by two-tailed Pearson correlation test. Pearson chi-square test was used to analyse patient blasts treatment response relative to disease characteristics. The level of significance was *p* < 0.05.

To calculate drug interaction, cell metabolic activity results were plotted into the interaction calculation software SynergyFinder+ [31], and Bliss independence score was used to calculate synergy. A positive synergy score indicates synergistic effect while a negative synergy score indicates antagonistic effect, and a score around zero suggests additive effect.

## Results

### Phenazine 5,10-dioxides with activity towards AML cell lines also induce cell death in AML patient blasts

To obtain a better understanding of the anti-AML efficacy of iodinin (Cpd **1**) and the most potent and AML selective derivatives [6,7], the compounds were tested for their ability to induce cell death on a panel of patient derived AML blasts. DNR was included for comparison of efficacy, and to correlate sensitivity to phenazine 5,10-dioxides. The blasts were collected from patients with a median age of 65 (range 17 to 92) and classified according to the French-American-British (FAB) system. Patient characteristics are summarised in Table 2 and listed for each patient in Supplementary Table 1.

**Table 2:** Patient characteristics and response to treatment with phenazine 5,10-dioxides. Known clinical and biological characterisations of the patients and its association to phenazine 5,10-dioxide response. P-values are from Pearson chi-square test. ^‡^One patient had both ITD and Asp835 mutations in *FLT3* and therefore appears twice. F: female, M: male, n.d.: not determined, FAB: French-American-British classification, *FLT3*: FMS-like tyrosine kinase, ITD: internal tandem duplication, *NPM-1*: Nucleophosmin-1, INS: insertion, wt: wild type. Good, intermediate or adverse cytogenetics after the European Leukaemia Net (ELN) 2022 classification (10). Response categorised as high (>50 % cell death), intermediate (10–50 % cell death) and none (<10 % cell death) from ≤2.5 µM of Cpd **3, 6** or **9**.

Patient characteristics		Response			
	Total	None	Intermediate	High	p-value
Total	61	3	28	30	
***Sex***					0.934
Female	26	1	12	13	
Male	34	2	15	17	
n.d.	1	0	1	0	
**Biological characteristics**					
***FAB classification***					0.096
M0	4	0	0	4	
M1	11	1	2	8	
M2	11	0	8	3	
M4	11	1	6	4	
M5	19	0	10	9	
n.d.	5	1	2	2	
***CD34 expression***					0.679
Negative (<20 %)	20	0	9	11	
Positive (>20 %)	36	3	16	17	
Biclonal, subpop, heterogenous	3	0	1	2	
n.d.	2	0	2	0	
***Cytogenetic risk category***					0.688
Favourable	4	0	3	1	
Intermediate	38	2	18	18	
Adverse	12	1	4	7	
n.d.	7	0	3	4	
***M*u*tations***					
*FLT3 mutations*					0.907
ITD	19	0	9^‡^	10	
Asp835	2	1	1^‡^	0	
wt	33	1	14	18	
n.d.	8	1	5	2	
*NPM-1 mutations*					0.542
INS	19	0	9	10	
wt	34	2	14	18	
n.d.	8	1	5	2	

**Fig. 1:**
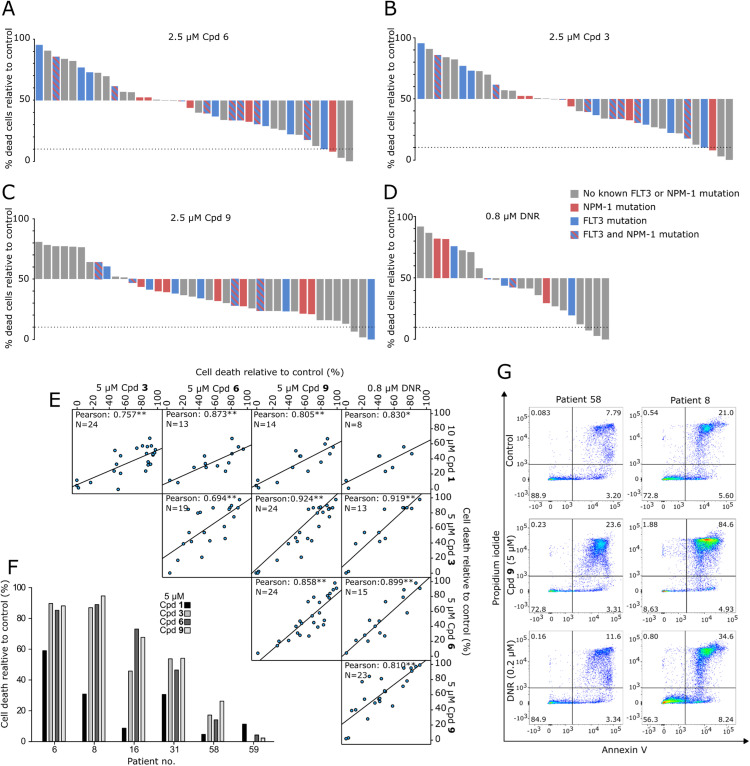
Patient derived AML blasts sensitivity to phenazine 5,10-dioxides. AML patient blasts were treated with Cpd **1, 3, 6, 9** or daunorubicin (DNR) for 24 h before evaluating cell death by flow cytometric analysis of cells stained with Annexin V and propidium iodide as described in the Methods section. The fraction of dead blasts was adjusted relative to control. A-D) Response of patient-derived blasts to Cpd **6** (A)**, 3** (B)**, 9** (C) and DNR (D) sorted according to response. Response illustrated above the x-axis is categorised as high (>50 % cell death), and response illustrated below the x-axis is categorised as intermediate (10–50 % cell death) or none (<10 % cell death, indicated by dashed line) from treatment with 2.5 µM of Cpd **3, 6** or **9**. Red, blue or striped columns indicate blasts with mutations in nucleophosmin-1 (*NPM-1*), FMS-like tyrosine kinase (*FLT3*) or both, respectively. E) Correlation between blasts response to Cpd **1, 3, 6, 9** and DNR. *p < 0.05, **p < 0.01, Two-sided Pearson correlation. F) Blasts isolated from six different AML patients were treated with 5 µM Cpd **1, 3, 6** or **9** for 24 h and viability assessed by flow cytometry. Data is given as percent increase in cell death relative to untreated. G) Flow cytometry scatter plots for patients 8 and 58 after treatment with 0.4 % DMSO (vehicle control), 5 µM Cpd **9** or 0.2 µM DNR.

The degree of response of the patient blasts was separated into high (>50 % cell death), intermediate (between 10–50 % cell death) and no response (<10 % cell death) after treatment with up to 2.5 µM of one of the phenazine 5,10-dioxides. A total of 30 patients were categorised as responders, 28 as intermediate responders and three patients were characterised as non-responders (Fig. 1A–D and Supplementary Figure 2). There was a positive linear correlation of the patient cells response to all tested phenazine 5,10-dioxides (Fig. 1C, *p* < 0.05). Cpd **9** had a very strong correlation to both Cpd **6** (Pearson coefficient 0.858, *p* < 0.01) and Cpd **3** (Pearson coefficient 0.924, *p* < 0.01). There was also correlation between the response to the phenazine 5,10 dioxides and DNR (Fig. 1E, Pearson correlation coefficient 0.919, 0.899 and 0.810 between DNR and Cpd **3, 6** or **9**, respectively, *p* < 0.01). Cpd **1** had the lowest potency of all the compounds towards the patient blasts (Fig. 1F shows results from selected responding and non-responding patients). Fig. 1G illustrates flow cytometry scatter plots for the low-responder patient (Pt) 58 and the high responder Pt 8 when left untreated, treated with 5 µM Cpd **9** and 0.2 µM DNR. No correlation was found between phenazine responders and the clinical and biological characterisations of the patients; sex, FAB-classification, cytogenetics, *FLT3* mutations, *NPM-1* insertion or CD34 expression (Table 2). The response of all patient blasts to the Phenazine 5,10-dioxide analogues and DNR can be found in Supplementary Figure 2.

Preliminary tests indicated a cytotoxic effect of Cpd **3** and **9** on BM cells which were defrosted from storage under liquid nitrogen (data not shown). However, due to difficulty in obtaining fresh BM aspirate, only a few compounds were tested, and further experiments could not be conducted.

### Phenazine 5,10-dioxide analogues exhibit differences in toxicity on zebrafish larvae

**Fig. 2:**
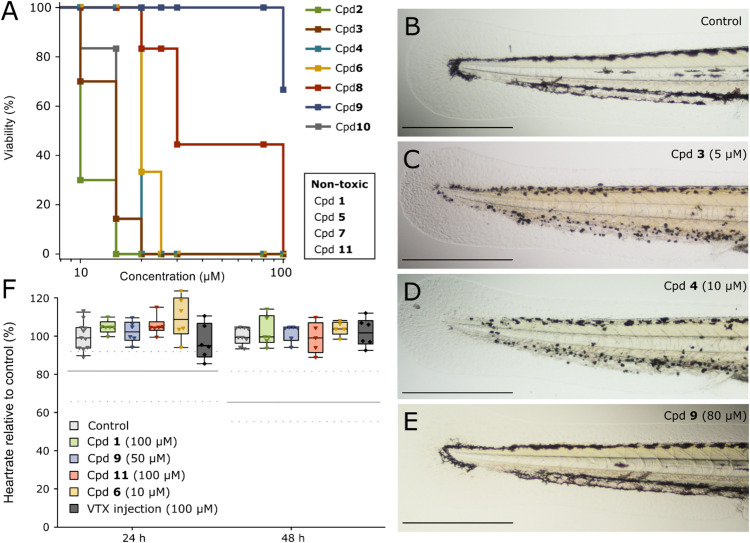
Toxicity of phenazine 5,10-dioxides and VTX in zebrafish larvae. A) Viability of zebrafish larvae after 48 h of exposure to various concentrations of phenazine 5,10-dioxide compounds. *n* = 3–9 per data point. B-E) Pigmentation abnormalities after treatment with the indicated compounds. Zebrafish larvae at 48 h post fertilisation were either incubated in embryo medium (control, B) or exposed to 5 µM Cpd **3** (C), 10 µM Cpd **4** (D) and 80 µM Cpd **9** (E), and imaged after 24 h of exposure. Scale bar: 500 µm. F) Heartrate of zebrafish larvae relative to control after treatment with the compounds or VTX for 24 and 48 h. VTX was intravenously injected (4 nL injection, 100 µM). No significant differences were detected in any groups, one-way ANOVA with Tukey’s honest significant difference post-hoc test, *n***=** 5–11. The grey horizontal line represents median heartrate (solid line) with standard deviation (dashed lines) from similar experiments with zebrafish larvae given an intravenous injection of 1 mM DNR [32].

Before analysing the efficacy of the compounds on xenograft AML cells in zebrafish larvae, it was necessary to test their toxicity on zebrafish larvae. The compounds were dissolved in the embryo medium, and viability of the larvae recorded over time. Cpd **2** and **3** were the most toxic compounds, killing zebrafish larvae already after six hours at 30 and 100 µM, respectively (data not shown). After 48 h exposure only 5 µM did not cause lethality (Fig. 2A). On the other hand, 48 h of treatment with either, Cpd **1, 5, 7** or **11** at 100 µM (the highest concentration tested) was not lethal to the zebrafish larvae. Precipitations of Cpd **5** were observed in the embryo medium from 30 to 100 µM, which could affect the exact concentration of compound available in the medium.

In wild-type AB-strain zebrafish larvae Cpd **2, 3, 4, 8** and **10** caused melanocyte abnormalities (Fig. 2B-D). This was not observed for Cpd **1, 6, 9** or **11**, illustrated in Fig. 2E for Cpd **9**. Anthracyclines, which are cardiotoxic in humans, reduce zebrafish larvae heartrate [32], and Cpd **1** is believed to have similar cytotoxic mechanism of action [5]. Furthermore, several phenazine 5,10-dioxides are cytotoxic towards H9c2, albeit at higher concentrations than MOLM-13 [6]. We therefore monitored the heartrate of zebrafish larvae exposed to phenazine 5,10-dioxides. However, no effects on heartrate were found relative to control (Fig. 2F). Toxicity and maximum tolerated doses (MTD) of the chemicals on zebrafish larvae are summarised in Table 3.

**Table 3:** Toxicity of phenazine 5,10-dioxides in zebrafish larvae. Maximum tolerated doses and observed morphological alterations in zebrafish larvae caused by phenazine 5,10-dioxides dissolved in embryo medium for 48 h. Cpd: compound. MTD: maximum tolerated dose. a: EC_50_ values from [6], –: not observed.

Cpd.	MOLM-13 EC_50_ (µM)	MTD (µM)	Pigmentation alterations	Muscle fibre alterations	Pericardial oedema
Iodinin **(1)**	2.0 ± 0.1^a^	>100	**–**	**–**	**–**
Myxin **(2)**	1.4 ± 0.3^a^	5	From 10 µM	**–**	**–**
**3**	0.57 ± 0.06^a^	<5	From 5 µM	**–**	**–**
**4**	0.89 ± 0.03^a^	5	From 10 µM	From 15 µM	From 15 µM
**5**	1.5 ± 0.2^a^	>100	**–**	**–**	**–**
**6**	0.34 ± 0.04^a^	15	**–**	**–**	**–**
**7**	1.0 ± 0.06^a^	>100	**–**	**–**	**–**
**8**	0.89 ± 0.06	<10	From 10 µM	**–**	**–**
**9**	0.63 ± 0.05^a^	50	**–**	**–**	From 80 µM
**10**	1.4 ± 0.1	<10	From 10 µM	From 10 µM	**–**
**11**	0.63 ± 0.05	>100	**–**	**–**	**–**

### Cpd 9 and 11 reduce MOLM-13 AML tumour burden in zebrafish larvae

Only the compounds that had a MTD in zebrafish larvae of at least 10 times the EC_50_-value in MOLM-13 cells were selected for further experiments, thus Cpd **2, 3, 4, 8** and **10** were excluded. Cpd **5** and **7** were included in the current work due to their high selectivity towards AML cells relative to cell lines derived from healthy tissue. However, contrary to the other compounds, they have low membrane permeability [6], which along with the observed precipitations and high tolerability in zebrafish larvae suggests minimal absorption through the skin. Consequently, Cpd **6, 9** and **11** were considered best suited for treatment of zebrafish larvae with xenograft AML cells.

**Fig. 3:**
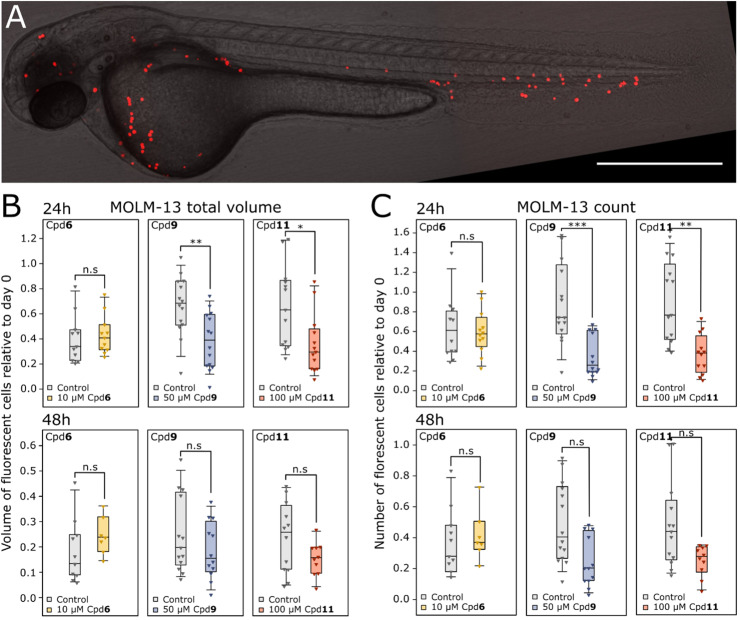
Anti-cancer efficacy of Cpd **6, 9** and **11** towards xenograft MOLM-13 cells in zebrafish larvae. Zebrafish larvae in the long pec stage were intravenously injected with CellTracker™ Deep Red stained MOLM-13 cells and left untreated (Control) or treated with Cpd **6** (10 µM), Cpd **9** (50 µM) or Cpd **11** (100 µM) by dissolution in the embryo medium. A) A zebrafish larva imaged two hours after injection with MOLM-13 cells (red). The scale bar represents 0.5 mm. B-C) The MOLM-13 total volume (B) and count (C) after 24 h of treatment relative to before treatment (day 0). *p < 0.05, **p < 0.01, ***p < 0.001, two-tailed, two-sample t-test. *n* = 7–14.

A zebrafish larva two hours after intravenous injection of fluorescently labelled MOLM-13 cells is illustrated in Fig. 3A with the transplanted cells visible in red. After 24 h of treatment, the larvae that were exposed to Cpd **9** or **11** showed a significant reduction in MOLM-13 cells compared to the control group (Fig. 3B and C). After 48 h there was still an apparent reduction compared to control, but this was not significant. The zebrafish larvae xenografts treated with Cpd **6** showed no response at 24 or 48 h compared to the control group.

### Cpd 1 and 9 act synergistically with venetoclax towards AML cell lines

In AML therapy, a combination of two or several drugs is shown to be more efficient than the use of a single drug alone. We performed a series of preliminary experiments with Cpd **1** in combination with several drugs used to treat AML like DNR, cytarabine and etoposide , but only detected additive effects when investigating drug interaction (data not shown). However, the response to VTX appeared to be potentiated by the presence of Cpd **1**. Since VTX-based treatment is the new standard for AML patients unfit for intensive chemotherapy, it was of interest to further investigate the combination of VTX with selected phenazine 5,10-dioxides.

**Fig. 4:**
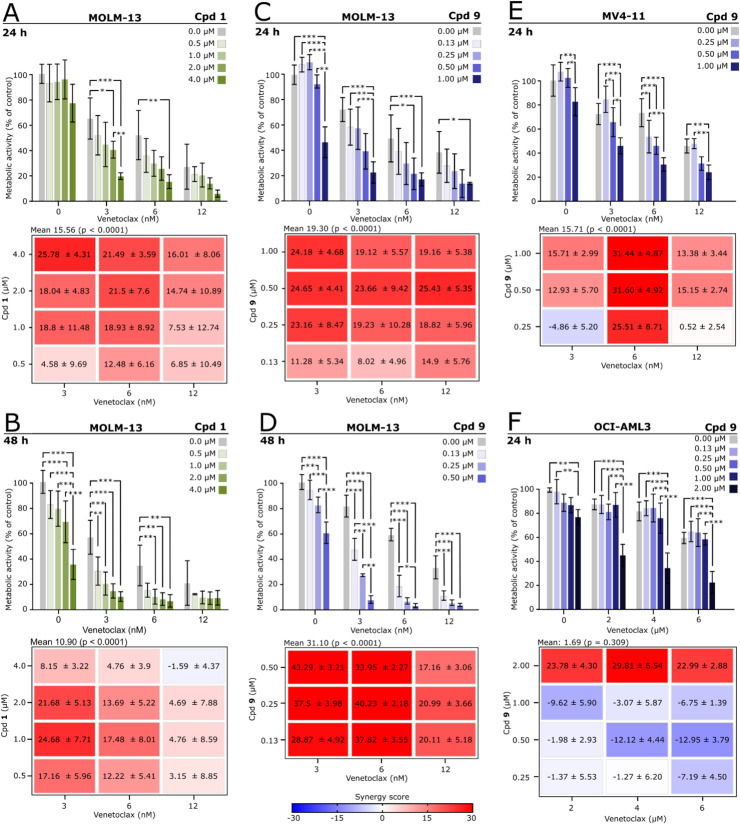
In vitro efficacy and synergy of phenazine 5,10-dioxides and venetoclax. MOLM-13, MV4–11 and OCI-AML3 cells were treated with Cpd **1** or **9** alone or in combination with VTX for 24 or 48 h before metabolic activity was determined by the WST-1 proliferation assay, and drug interactions were calculated using Bliss independence in the SynergyFinder+ tool. A) MOLM-13 cells treated with Cpd **1** and VTX for 24 h. B) MOLM-13 cells treated with Cpd **1** and VTX for 48 h. C) MOLM-13 cells treated with Cpd **9** and VTX for 24 h. D) MOLM-13 cells treated with Cpd **9** and VTX for 48 h. E) MV4–11 cells treated with Cpd **9** and VTX for 24 h. F) OCI-AML3 cells treated with Cpd **9** and VTX for 24 h. *p < 0.05, **p < 0.01, ***p < 0.001, One-way ANOVA with Tukey’s honest significant difference post-hoc, *n* = 3–6.

We found that a sub-toxic concentration of Cpd **1** increased the cytotoxic response of MOLM-13 cells treated with VTX after 24 h of incubation (Fig. 4A), and that this synergistic effect was also present after 48 h of incubation (Fig. 4B). The mean Bliss interaction indexes for all tested combinations of drug concentrations of Cpd **1** and VTX were 15.56 for 24 h and 10.90 for 48 h (*p* < 0.001) suggesting a strong synergistic effect.

For the analogue Cpd **9**, there was an even higher synergistic drug interaction with VTX, again with non-toxic concentrations of Cpd **9** (Fig. 4C and D). For instance, 0.5 µM Cpd **9** and 6 nM VTX alone reduced respective metabolic activity to 60 % and 59 %, but the combination treatment caused only 5 % residual relative metabolic activity after 48 h (Fig. 4D). There was synergism between all tested concentrations of Cpd **9** and VTX with a mean Bliss interaction index of 19.30 for 24-hour incubation (Fig. 4C, *p* < 0.001) and 31.10 for 48-hour incubation (Fig. 4D, *p* < 0.001).

The synergistic drug interaction between Cpd **9** and VTX was also present in MV4–11 cells after 24 h of incubation (Fig. 4E). Here, 1 µM Cpd **9** did not reduce metabolic activity alone (*p* = 0.086, Fig. 4E), but there was a synergistic effect with all tested combinations of Cpd **9** and VTX except for the lowest concentration of Cpd **9** and 3 or 12 nM VTX. The overall Bliss independence index was 15.71 (Fig. 4E, *p* < 0.001).

The OCI-AML3 cells showed high resistance to VTX, with no reduction in metabolic activity until 6 µM, a thousand-fold higher than for MOLM13 and MV4–11 (Fig. 4F). Interestingly, a synergistic effect with all tested concentrations of VTX was observed in combination with 2 µM Cpd **9**, the highest tested concentration for the compound (Fig. 4F). Due to the lack of synergy at the lower concentrations, however, the mean Bliss independence index was not significant (1.67, *p* = 0.309, Fig. 4F).

All statistics from metabolic activity that are not illustrated in Fig. 4 can be found in Supplementary Tables 2–7.

### Both Cpd 9 and VTX reduce cancer cell burden in zebrafish larvae

**Fig. 5:**
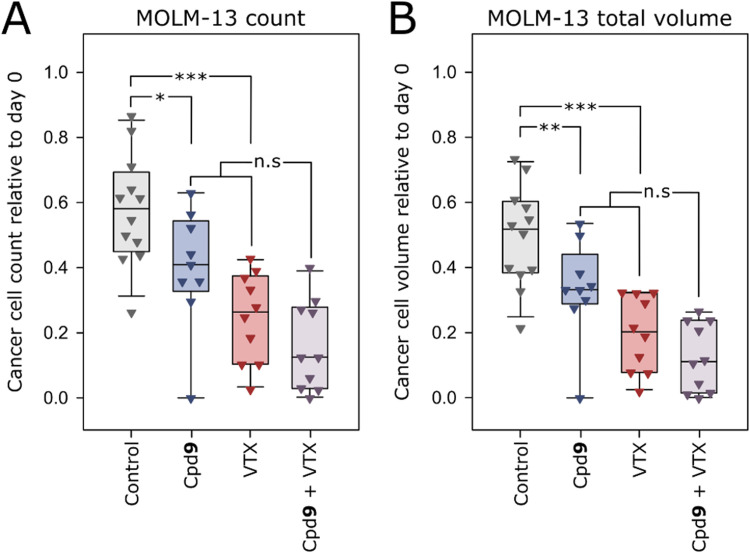
Interaction effect of Cpd 9 and VTX towards MOLM-13 cells in zebrafish larvae. Zebrafish larvae in the long pec stage were intravenously injected with CellTracker™ Deep Red dyed MOLM-13 cells at 2 dpf and left untreated or treated with Cpd **9** (50 µM dissolved in the embryo medium), VTX (4 nL 50 µM intravenous injection) or a combination of the two. Xenograft MOLM-13 count (A) and total volume (B) were quantified prior to treatment and after 24 h of treatment. Two-way ANOVA: *p < 0.05, **p < 0.01, ***p < 0.001, *n* = 9–12.

Since a synergistic effect between phenazine 5,10-dioxides and VTX was observed in vitro towards MOLM-13-cells, we wanted to test whether a similar effect could be observed in zebrafish larvae with intravenously injected MOLM-13 cells. Due to poor solubility, VTX had to be intravenously injected while Cpd **9** was administered in the embryo medium. Both VTX and Cpd **9** reduced cancer cell burden relative to control (*p* < 0.001 for VTX and *p* < 0.05 for Cpd **9**), and while there was a trend towards an enhanced effect with the combination relative to the monotherapy, it was not significant (*p* = 0.360 for MOLM-13 count and *p* = 0.301 for MOLM-13 total volume, Fig. 5).

## Discussion

In the present study, phenazine 5,10-dioxide compounds demonstrated high cytotoxicity towards patient derived AML blasts (Fig. 1). We did not find any correlation with the blast's sensitivity to the phenazine 5,10-dioxides based on the known biological characteristics of the patient blasts, such as adverse cytogenetic profile, CD34 expression and *FLT3*-ITD or *NPM-1* mutations, (Table 1, Supplementary Table 1). Interestingly, the blasts from both Pt 11 and Pt 20 had all analysed markers of poor prognosis, namely adverse cytogenetics, *FLT3*-ITD, CD34 positive, and wild-type *NPM-1* [10,33,34] but were both in the responder group (Fig. 1, Supplementary Table 1 and Supplementary Figure 2). Monocytic differentiation is associated with clinical resistance to the BCL2 inhibitor venetoclax, probably because monocytic FAB-M4/M5 cells show relatively low BCL2 levels and rather rely on Mcl-1 for metabolic regulation and survival (for a detailed discussion and references see [35]. However, FAB-M4/M5 AML cells did not differ from the other patients regarding susceptibility phenazine 5,10-dioxides analogues (Fisher’s exact test, *p* = 0.40). Thus, the effect of the analogues seems independent of these differences in the expression and function of BCL2/MCL1.

Taken together, these results indicates that the compounds should be further investigated as a treatment option for patients with poor prognostic characteristics. Since there was a high correlation between the response to the phenazine 5,10-dioxides and DNR (Fig. 1C), the former could represent an alternative treatment to DNR in patients that are unfit for intensive therapy. The cytotoxic effect of DNR is believed to be due to DNA-intercalation, inhibition of topoisomerase IIα and generation of free radicals [36], and several phenazines including Cpd **1** and **2** have been known to bind DNA and inhibit DNA replication or RNA synthesis [37,38]. For instance, Cpd **2** is reported to cause DNA strand cleavage where the loss of oxygen from the *N*-oxide groups were important also under hypoxic conditions [39]. In line with this, we have shown that analogues where the *N*-oxide is missing have up to a hundred-fold lower cytotoxic activity [7]. However, we cannot exclude other modes of action like direct activity modulation on signalling proteins, and this should be further investigated in future studies. We have previously shown that anthracyclines reduced the heart rate of zebrafish larvae [32,40], but none of the phenazine 5,10-dioxides tested affected heart rate (Fig. 2F). This could indicate a more selective effect of phenazine 5,10-dioxides towards cancer cells relative to healthy tissue, which would be beneficial for patients who are ineligible for intensive chemotherapy with anthracyclines due to high risk of toxic side effects. In line with this, we found that Cpd **9** and **11** both significantly reduced the tumour burden of xenograft MOLM-13 cells in zebrafish larvae after 24 h treatment (Fig. 3B and C).

We previously identified Cpd **6** as one of the most promising phenazine 5,10-dioxides (Cpd. **54** in [6]) with an EC_50_ as low as 0.34 µM towards MOLM-13 cells and low toxicity towards the non-cancerous kidney epithelial NRK cells. However, the cardiomyoblast cell line H9c2 was more responsive than the NRK cells, and in accordance, a low MTD on zebrafish larvae was found for Cpd **6**, albeit without signs of cardiotoxicity (Table 2, Fig. 2F). Due to the low MTD of Cpd **6** compared to Cpd **9** and **11,** the therapeutic range was thus much narrower and it was administered in respective five- and tenfold lower concentration to avoid toxic effects. Accordingly, no effect of Cpd **6** was observed for tumour burden in zebrafish larvae. These data highlight the usefulness of a model system which rapidly detects unwanted effects of a therapy. The zebrafish larva is ideal to exclude compounds that show potential in cell experiments, but would fail in more advanced animal models, as demonstrated in other studies on novel AML drugs [41,42].

Both Cpd **1** and **9** acted synergistic with VTX against the three cell lines MOLM-13, MV4–11 and OCI-AML3 (Fig. 4). However, for OCI-AML3 the interaction was not apparent until high concentrations of both drugs. The concentration of VTX needed to induce cell death were above what is reported as the plasma concentrations in patients receiving high dosage of VTX. The reported C_max_ of patients receiving 400 mg VTX as a single dosage was around 2.5 µM, whereas the steady state concentration is between 1 and 5 µM (43). Even though 2 µM of VTX strongly potentiated the effect of Cpd **9**, it can be argued whether such drug concentrations can be obtained in the leukaemic bone marrow. Others have also found that even though VTX act synergistic with other drugs such as the cyclin-dependent kinase inhibitor avolcidib against OCI-AML3, the cell line tolerates high VTX concentrations both alone and in combination compared to MOLM-13 and MV4–11, and is regarded resistant [44,45]. This resistance is likely connected to a high expression of survival proteins like Bcl-2 and MCL-1 in the OCI-AML3 cells compared to MOLM-13 or MV4–11 [46]. Downregulation of these factors can restore VTX response in resistant AML cells [47]. Still, the phenazine 5,10-dioxide did not fully restore VTX-response, pointing towards a mode of action not related to attenuation of antiapoptotic proteins.

Several AML drugs have shown to synergise with VTX, both conventional agents like hypomethylating or chemotherapeutic agents (see [48] for a review) and new targeted therapies like FLT3 inhibitors [49] or MCL1 inhibitors [50]. Synergism with VTX appears to be somewhat independent of the mechanism of action of the added drugs, but still highly relevant, since resistance towards VTX has been reported as a concern [51].

A trend towards reduced tumour burden in zebrafish larvae was observed for Cpd **9** and VTX in combination, however no statistically significant effect was present relative to treatment with the two drugs alone (Fig. 5). A highly potent effect of VTX alone seem to partially explain the outcome. Additionally, the larvae are kept at 31.5 °C, a sub-optimal environment for human cancer cells resulting in a gradual reduction in tumour burden also in the untreated control groups (Fig. 3, Fig. 5). It would be of interest to study if the anti-leukemic effect persists in larvae also after removal of the drug, and at incubation times of >48 h. The combination effect could perhaps be more apparent after long-time experiments, but further optimisation and validation of the model is required to undertake such studies. However, the synergistic effect of VTX and Cpd **9** in vitro for MOLM-3 and MV4–11 indicates that the phenazine 5,10-dioxides could be beneficial in combination with VTX for patients with VTX-responsive AML.

To summarise, we present further results demonstrating the potential of phenazine 5,10-dioxides in AML treatment. Several AML model systems, including AML patient blasts, cell lines, and zebrafish larvae transplanted with AML cells confirm our preliminary findings from previous publications [6,7]. Importantly, the phenazine 5,10-dioxides have potent synergy with the AML drug VTX, indicating that these compounds could be future treatment options for AML patients who today are considered unfit for intensive chemotherapy.

## CRediT authorship contribution statement

**Ingeborg Nerbø Reiten:** Writing – review & editing, Writing – original draft, Visualization, Validation, Methodology, Investigation, Formal analysis, Data curation, Conceptualization. **Reidun Aesoy:** Methodology, Writing – review & editing, Writing – original draft, Validation, Data curation. **Jan-Lukas Førde:** Methodology, Investigation, Formal analysis. **Goraksha Machhindra Khose:** Resources, Investigation. **Elvar Örn Viktorsson:** Writing – review & editing, Resources, Project administration, Methodology. **Øystein Bruserud:** Resources, Methodology, Data curation. **Pål Rongved:** Resources, Project administration, Methodology, Funding acquisition. **Håkon Reikvam:** Writing – review & editing, Supervision, Resources, Project administration, Methodology, Funding acquisition. **Lars Herfindal:** Writing – review & editing, Writing – original draft, Validation, Supervision, Resources, Project administration, Methodology, Investigation, Funding acquisition, Formal analysis, Conceptualization.

## Declaration of competing interest

The authors declare that they have no known competing financial interests or personal relationships that could have appeared to influence the work reported in this paper.

## Appendix Supplementary materials

Image, application 1
