# Supplementary material for: Phenazine 5,10-dioxide analogues as potential therapeutics in AML: Efficacy on patient-derived blasts, in zebrafish larvae xenografts and synergy with venetoclax
Source: Transl Oncol. 2025 Dec 6;63:102628. doi: 10.1016/j.tranon.2025.102628 (PMC12741294; doi:10.1016/j.tranon.2025.102628)
Supplement: Supplementary file 1 [file mmc1.docx]

# Supplementary Information accompanying the manuscript

# “Phenazine 5,10-dioxide analogues as potential therapeutics in AML: Efficacy on patient-derived blasts, in zebrafish larvae xenografts and synergy with venetoclax”

Ingeborg Nerbø Reiten^1^, Reidun Æsøy^1^, Jan-Lukas Førde^1^, Goraksha Machhindra Khose^2^, Elvar Örn Viktorsson^2^, Øystein Bruserud^4,5^, Pål Rongved^3^, Håkon Reikvam^4,5^, Lars Herfindal^1*^.

1: Centre for Pharmacy, Department of Clinical Science, University of Bergen

2: School of Health Sciences, Faculty of Pharmaceutical Sciences, University of Iceland

3: School of Pharmacy, Department of Pharmaceutical Chemistry, University of Oslo

4: Department of Medicine Haukeland University Hospital, Bergen, Norway

5: K.G. Jebsen Center for Myeloid Malignancies, Department of Clinical Science, University of Bergen, Bergen, Norway

* Corresponding author: lars.herfindal@uib.no

**Supplementary Table 1: Patient blast characteristics.** F: female, M: male, n.d.: not determined, +: positive (>20%), -: negative (<20%), FAB: French-American-British classification, *FLT3*: FMS-like tyrosine kinase, ITD: internal tandem duplication, *NPM-1*: Nucleophosmin-1, INS: insertion, wt: wild type, Pt: patient. Good, intermediate or adverse cytogenetics after the European Leukaemia Net (ELN) 2022 classification (DOI: Blood (2022) 140 (12): 1345–1377).

| Pt | Sex | Age | Fab | CD34 | Cytogen | FLT3 | NPM-1 |
| --- | --- | --- | --- | --- | --- | --- | --- |
| 1 | F | 92 | M1 | - | n.d. | n.d. | n.d. |
| 2 | F | 75 | M1 | + | n.d. | ITD | wt |
| 3 | M | 78 | M0 | - | Adverse | wt | wt |
| 4 | F | 77 | M1/2 | heterogenous | Intermediate | ITD | INS |
| 5 | F | 87 | M0 | + | Adverse | wt | wt |
| 6 | M | 68 | M1 | + | Intermediate | wt | wt |
| 7 | M | 67 | M1 | + | Intermediate | ITD | wt |
| 8 | M | 65 | M5 | subpop | Good | wt | wt |
| 9 | F | 75 | M4 | + | Intermediate | ITD | wt |
| 10 | M | 82 |  | + | Intermediate | wt | wt |
| 11 | F | 64 | M1 | + | Adverse | ITD | wt |
| 12 | M | 60 | M4 | + | Intermediate | ITD | wt |
| 13 | F | 63 | M1 | + | Intermediate | wt | wt |
| 14 | M | 60 | M2 | + | Intermediate | n.d. | n.d. |
| 15 | M | 86 | M5 | - | Intermediate | wt | wt |
| 16 | M | 48 | M5 | - | Intermediate | ITD | INS |
| 17 | M | 41 | M4 | + | Intermediate | wt | wt |
| 18 | F | 59 | M5 | - | Intermediate | ITD | INS |
| 19 | M | 42 | M5 | + | Intermediate | ITD | INS |
| 20 | M | 77 | M2 | + | Adverse | ITD | wt |
| 21 | M | 54 | M5 | - | Intermediate | wt | INS |
| 22 | M | 78 | M1 | + | Intermediate | wt | wt |
| 23 | M | 65 | M5 | - | Adverse | wt | INS |
| 24 | M | 72 | M5 | - | Intermediate | wt | INS |
| 25 | M | 76 | M5 | + | Adverse | wt | wt |
| 26 | F | 77 | M1 | - | n.d. | wt | INS |
| 27 | F | 17 | M2 | + | Intermediate | wt | wt |
| 28 | F | 71 | M0 | - | Intermediate | wt | INS |
| 29 | F | 51 | M0 | + | Adverse | wt | wt |
| 30 | F | 70 | M4 | - | n.d. | wt | INS |
| 31 | F | 49 | M2 | + | Adverse | n.d. | n.d. |
| 32 | F | 74 | M4 | + | Good | ITD | INS |
| 33 | F | 29 | M5 | + | Intermediate | ITD Asp835 | wt |
| 34 | M | 46 | M1 | n.d. | Intermediate | wt | INS |
| 35 | F | 82 | M2 | + | Intermediate | ITD | INS |
| 36 | M | 36 | M5 | + | Good | ITD | wt |
| 37 | M | 36 | M4 | + | Good | wt | wt |
| 38 | M | 72 | M4 | - | Intermediate | n.d. | n.d. |
| 39 | M | 47 | M5 | + | Adverse | wt | wt |
| 40 | M | 59 | M5 | + | Intermediate | ITD | wt |
| 41 | M | 32 | M2 | + | Intermediate | wt | wt |
| 42 | F | 59 | M4 | - | Intermediate | ITD | INS |
| 43 |  | 46 | M1 | - | Intermediate | wt | INS |
| 44 | F | 55 | M5 | - | Intermediate | ITD | INS |
| 45 | M | 71 | M4/5 | n.d. | n.d. | n.d. | n.d. |
| 46 | F | 80 | M2 | + | n.d. | n.d. | n.d. |
| 47 | F | 64 | M4 | + | Adverse | wt | wt |
| 48 | M | 35 | M2 | + | Intermediate | wt | wt |
| 49 | F | 46 | M2 | + | Intermediate | wt | wt |
| 50 | M | 20 | M2 | + | Intermediate | ITD | wt |
| 51 | F | 68 | M5 | - | Intermediate | wt | INS |
| 52 | M | 64 | M5 | - | Intermediate | wt | INS |
| 53 | M | 19 | M5 | - | Intermediate | wt | wt |
| 54 | F | 75 | M2 | + | n.d. | n.d. | n.d. |
| 55 | M | 79 | M4 | biklonal | Intermediate | wt | wt |
| 56 | M | 65 | M5 | - | Intermediate | ITD | INS |
| 57 | M | 60 | M5 | - | Adverse | wt | wt |
| 58 | F | 85 |  | + | Intermediate | wt | wt |
| 59 | M | 68 | M4 | + | Intermediate | Asp835 | wt |
| 60 | M | 78 | M1 | + | Adverse | n.d. | n.d. |
| 61 | F | 71 |  | + | Intermediate | wt | wt |


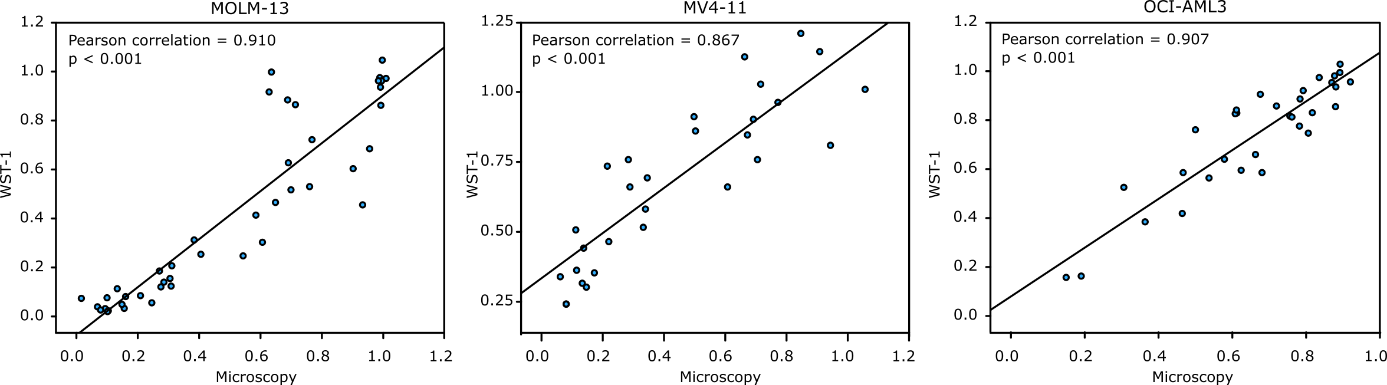


**Supplementary Figure 1:** Correlation of viability between WST-1 and microscopy analyses.

**
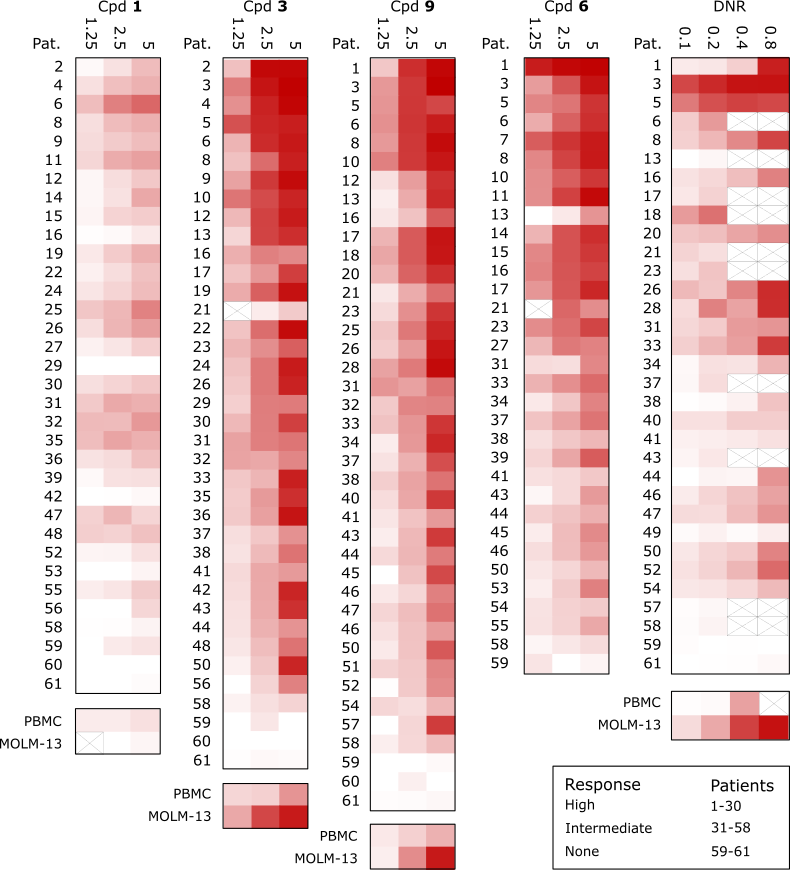
**

**Supplementary Figure 2:** Primary AML blast’s response to treatment with phenazine 5,10-dioxides and DNR. Blasts were treated with Cpd **1**, **3**, **6**, **9** or daunorubicin (DNR) for 24 hours before evaluating cell death by flow cytometric analysis of cells stained with Annexin V and propidium iodide as described in the Methods section. The fraction of dead blasts was adjusted relative to control. Response categorised as high (>50% cell death from ≤2.5 µM of Cpd **3**, **6** or **9**), intermediate (10-50% cell death from ≤2.5 µM of Cpd **3**, **6** or **9**) and none (<10% cell death from ≤2.5 µM of Cpd **3**, **6** or **9**). PBMC: peripheral blood mononuclear cells.

**Supplementary Table 2:** Statistics from viability of MOLM-13 cells treated with iodinin (Cpd**1**) and VTX for 24 hours. P-values were calculated using one-way ANOVA with Tukey’s HSD post-hoc test. Significant values p≤0.05 are highlighted with red numbers. N=3-6

| **0 nM VTX** | **0 µM Cpd1** | **0.5 µM Cpd1** | **1.0 µM Cpd1** | **2.0 µM Cpd1** |  | **0 µM Cpd1** | **0 nM VTX** | **3 nM VTX** | **6 nM VTX** |
| --- | --- | --- | --- | --- | --- | --- | --- | --- | --- |
| **4 µM Cpd1** | 0.082 | 0.877 | 0.810 | 0.655 |  | **12 nM VTX** | 0.000 | 0.009 | 0.393 |
| **2.0 µM Cpd1** | 1.000 | 1.000 | 1.000 |  |  | **6 nM VTX** | 0.000 | 0.978 |  |
| **1.0 µM Cpd1** | 1.000 | 1.000 |  |  |  | **3 nM VTX** | 0.000 |  |  |
| **0.5 µM Cpd1** | 1.000 |  |  |  |  |  |  |  |  |
|  |  |  |  |  |  |  |  |  |  |
| **3 nM VTX** | **0 µM Cpd1** | **0.5 µM Cpd1** | **1.0 µM Cpd1** | **2.0 µM Cpd1** |  | **0.5 µM Cpd1** | **0 nM VTX** | **3 nM VTX** | **6 nM VTX** |
| **4 µM Cpd1** | 0.000 | 0.012 | 0.164 | 0.458 |  | **12 nM VTX** | 0.000 | 0.102 | 0.983 |
| **2.0 µM Cpd1** | 0.188 | 0.995 | 1.000 |  |  | **6 nM VTX** | 0.000 | 0.871 |  |
| **1.0 µM Cpd1** | 0.501 | 1.000 |  |  |  | **3 nM VTX** | 0.000 |  |  |
| **0.5 µM Cpd1** | 0.975 |  |  |  |  |  |  |  |  |
|  |  |  |  |  |  |  |  |  |  |
| **6 nM VTX** | **0 µM Cpd1** | **0.5 µM Cpd1** | **1.0 µM Cpd1** | **2.0 µM Cpd1** |  | **1.0 µM Cpd1** | **0 nM VTX** | **3 nM VTX** | **6 nM VTX** |
| **4 µM Cpd1** | 0.002 | 0.479 | 0.941 | 0.998 |  | **12 nM VTX** | 0.000 | 0.449 | 1.000 |
| **2.0 µM Cpd1** | 0.107 | 0.999 | 1.000 |  |  | **6 nM VTX** | 0.000 | 0.923 |  |
| **1.0 µM Cpd1** | 0.337 | 1.000 |  |  |  | **3 nM VTX** | 0.000 |  |  |
| **0.5 µM Cpd1** | 0.862 |  |  |  |  |  |  |  |  |
|  |  |  |  |  |  |  |  |  |  |
| **12 nM VTX** | **0 µM Cpd1** | **0.5 µM Cpd1** | **1.0 µM Cpd1** | **2.0 µM Cpd1** |  | **2.0 µM Cpd1** | **0 nM VTX** | **3 nM VTX** | **6 nM VTX** |
| **4 µM Cpd1** | 0.844 | 0.992 | 0.996 | 1.000 |  | **12 nM VTX** | 0.000 | 0.285 | 0.999 |
| **2.0 µM Cpd1** | 0.999 | 1.000 | 1.000 |  |  | **6 nM VTX** | 0.000 | 0.925 |  |
| **1.0 µM Cpd1** | 1.000 | 1.000 |  |  |  | **3 nM VTX** | 0.000 |  |  |
| **0.5 µM Cpd1** | 1.000 |  |  |  |  |  |  |  |  |
|  |  |  |  |  |  |  |  |  |  |
|  |  |  |  |  |  | **4 µM Cpd1** | **0 nM VTX** | **3 nM VTX** | **6 nM VTX** |
|  |  |  |  |  |  | **12 nM VTX** | 0.000 | 0.992 | 1.000 |
|  |  |  |  |  |  | **6 nM VTX** | 0.000 | 1.000 |  |
|  |  |  |  |  |  | **3 nM VTX** | 0.000 |  |  |

**Supplementary table 3:** Statistics from viability of MOLM-13 cells treated with iodinin (Cpd **1**) and VTX after 48 hours treatment. P-values were calculated using one-way ANOVA with Tukey’s HSD post-hoc test. Significant values ≤0.05 are highlighted with red numbers. N=3-6

| **0 nM VTX** | **0 µM Cpd1** | **0.5 µM Cpd1** | **1.0 µM Cpd1** | **2.0 µM Cpd1** |  | **0 µM Cpd1** | **0 nM VTX** | **3 nM VTX** | **6 nM VTX** |
| --- | --- | --- | --- | --- | --- | --- | --- | --- | --- |
| **4 µM Cpd1** | 0.000 | 0.000 | 0.000 | 0.000 |  | **12 nM VTX** | 0.000 | 0.000 | 0.757 |
| **2.0 µM Cpd1** | 0.000 | 0.590 | 0.936 |  |  | **6 nM VTX** | 0.000 | 0.017 |  |
| **1.0 µM Cpd1** | 0.082 | 1.000 |  |  |  | **3 nM VTX** | 0.000 |  |  |
| **0.5 µM Cpd1** | 0.383 |  |  |  |  |  |  |  |  |
|  |  |  |  |  |  |  |  |  |  |
| **3 nM VTX** | **0 µM Cpd1** | **0.5 µM Cpd1** | **1.0 µM Cpd1** | **2.0 µM Cpd1** |  | **0.5 µM Cpd1** | **0 nM VTX** | **3 nM VTX** | **6 nM VTX** |
| **4 µM Cpd1** | 0.000 | 0.061 | 0.953 | 1.000 |  | **12 nM VTX** | 0.000 | 0.321 | 1.000 |
| **2.0 µM Cpd1** | 0.000 | 0.367 | 1.000 |  |  | **6 nM VTX** | 0.000 | 0.440 |  |
| **1.0 µM Cpd1** | 0.000 | 0.956 |  |  |  | **3 nM VTX** | 0.000 |  |  |
| **0.5 µM Cpd1** | 0.001 |  |  |  |  |  |  |  |  |
|  |  |  |  |  |  |  |  |  |  |
| **6 nM VTX** | **0 µM Cpd1** | **0.5 µM Cpd1** | **1.0 µM Cpd1** | **2.0 µM Cpd1** |  | **1.0 µM Cpd1** | **0 nM VTX** | **3 nM VTX** | **6 nM VTX** |
| **4 µM Cpd1** | 0.001 | 0.992 | 1.000 | 1.000 |  | **12 nM VTX** | 0.000 | 0.971 | 1.000 |
| **2.0 µM Cpd1** | 0.002 | 0.999 | 1.000 |  |  | **6 nM VTX** | 0.000 | 0.941 |  |
| **1.0 µM Cpd1** | 0.005 | 1.000 |  |  |  | **3 nM VTX** | 0.000 |  |  |
| **0.5 µM Cpd1** | 0.084 |  |  |  |  |  |  |  |  |
|  |  |  |  |  |  |  |  |  |  |
| **12 nM VTX** | **0 µM Cpd1** | **0.5 µM Cpd1** | **1.0 µM Cpd1** | **2.0 µM Cpd1** |  | **2.0 µM Cpd1** | **0 nM VTX** | **3 nM VTX** | **6 nM VTX** |
| **4 µM Cpd1** | 0.990 | 1.000 | 1.000 | 1.000 |  | **12 nM VTX** | 0.000 | 1.000 | 1.000 |
| **2.0 µM Cpd1** | 0.987 | 1.000 | 1.000 |  |  | **6 nM VTX** | 0.000 | 1.000 |  |
| **1.0 µM Cpd1** | 0.992 | 1.000 |  |  |  | **3 nM VTX** | 0.000 |  |  |
| **0.5 µM Cpd1** | 1.000 |  |  |  |  |  |  |  |  |
|  |  |  |  |  |  |  |  |  |  |
|  |  |  |  |  |  | **4 µM Cpd1** | **0 nM VTX** | **3 nM VTX** | **6 nM VTX** |
|  |  |  |  |  |  | **12 nM VTX** | 0.015 | 1.000 | 1.000 |
|  |  |  |  |  |  | **6 nM VTX** | 0.000 | 1.000 |  |
|  |  |  |  |  |  | **3 nM VTX** | 0.004 |  |  |

**Supplementary Table 4:** Statistics from viability of MOLM-13 cells treated with Cpd **9** and VTX after 24 hours treatment. P-values were calculated using one-way ANOVA with Tukey’s HSD post-hoc test. Significant values ≤0.05 are highlighted with red numbers. N=3-6

| **0 nM VTX** | **0 nM Cpd9** | **125 nM Cpd9** | **250 nM Cpd9** | **500 nM Cpd9** |  | **0 nM Cpd9** | **0 nM VTX** | **3 nM VTX** | **6 nM VTX** |
| --- | --- | --- | --- | --- | --- | --- | --- | --- | --- |
| **1000 nM Cpd9** | 0.000 | 0.000 | 0.000 | 0.001 |  | **12 nM VTX** | 0.000 | 0.001 | 0.990 |
| **500 nM Cpd9** | 1.000 | 1.000 | 0.993 |  |  | **6 nM VTX** | 0.000 | 0.151 |  |
| **250 nM Cpd9** | 0.947 | 1.000 |  |  |  | **3 nM VTX** | 0.005 |  |  |
| **125 nM Cpd9** | 1.000 |  |  |  |  |  |  |  |  |
|  |  |  |  |  |  |  |  |  |  |
| **3 nM VTX** | **0 nM Cpd9** | **125 nM Cpd9** | **250 nM Cpd9** | **500 nM Cpd9** |  | **125 nM Cpd9** | **0 nM VTX** | **3 nM VTX** | **6 nM VTX** |
| **1000 nM Cpd9** | 0.000 | 0.001 | 0.001 | 0.156 |  | **12 nM VTX** | 0.000 | 0.011 | 0.997 |
| **500 nM Cpd9** | 0.079 | 0.993 | 0.976 |  |  | **6 nM VTX** | 0.000 | 0,431 |  |
| **250 nM Cpd9** | 0.962 | 1.000 |  |  |  | **3 nM VTX** | 0.000 |  |  |
| **125 nM Cpd9** | 0.909 |  |  |  |  |  |  |  |  |
|  |  |  |  |  |  |  |  |  |  |
| **6 nM VTX** | **0 nM Cpd9** | **125 nM Cpd9** | **250 nM Cpd9** | **500 nM Cpd9** |  | **0.25 µM Cpd9** | **0 nM VTX** | **3 nM VTX** | **6 nM VTX** |
| **1000 nM Cpd9** | 0.000 | 0.063 | 0.377 | 0.998 |  | **12 nM VTX** | 0.000 | 0.002 | 1.000 |
| **500 nM Cpd9** | 0.047 | 0.788 | 0.996 |  |  | **6 nM VTX** | 0.000 | 0.046 |  |
| **250 nM Cpd9** | 0.782 | 1.000 |  |  |  | **3 nM VTX** | 0.000 |  |  |
| **125 nM Cpd9** | 0.996 |  |  |  |  |  |  |  |  |
|  |  |  |  |  |  |  |  |  |  |
| **12 nM VTX** | **0 nM Cpd9** | **125 nM Cpd9** | **250 nM Cpd9** | **500 nM Cpd9** |  | **0.5 µM Cpd9** | **0 nM VTX** | **3 nM VTX** | **6 nM VTX** |
| **1000 nM Cpd9** | 0.011 | 0.350 | 0.607 | 1.000 |  | **12 nM VTX** | 0.000 | 0.006 | 1.000 |
| **500 nM Cpd9** | 0.253 | 0.975 | 0.998 |  |  | **6 nM VTX** | 0.000 | 0.094 |  |
| **250 nM Cpd9** | 0.982 | 1.000 |  |  |  | **3 nM VTX** | 0.000 |  |  |
| **125 nM Cpd9** | 0.999 |  |  |  |  |  |  |  |  |
|  |  |  |  |  |  |  |  |  |  |
|  |  |  |  |  |  | **1 µM Cpd9** | **0 nM VTX** | **3 nM VTX** | **6 nM VTX** |
|  |  |  |  |  |  | **12 nM VTX** | 0.000 | 0.781 | 1.000 |
|  |  |  |  |  |  | **6 nM VTX** | 0.000 | 0.991 |  |
|  |  |  |  |  |  | **3 nM VTX** | 0.000 |  |  |

**Supplementary Table 5:** Statistics from viability of MOLM-13 cells treated with Cpd **9** and VTX after 48 hours. P-values were calculated using one-way ANOVA with Tukey’s HSD post-hoc test. Significant values ≤0.05 are highlighted with red numbers. N=3-6

| **0 nM VTX** | | **0 nM Cpd9** | | **125 nM Cpd9** | | **250 nM Cpd9** |  | **0 nM Cpd9** | **0 nM VTX** | **3 nM VTX** | **6 nM VTX** |
| --- | --- | --- | --- | --- | --- | --- | --- | --- | --- | --- | --- |
| **500 nM Cpd9** | | 0.000 | | 0.000 | | 0.000 |  | **12 nM VTX** | 0.000 | 0.000 | 0.000 |
| **250 nM Cpd9** | | 0.002 | | 0.309 | |  |  | **6 nM VTX** | 0.000 | 0.000 |  |
| **125 nM Cpd9** | | 0.956 | |  | |  |  | **3 nM VTX** | 0.001 |  |  |
|  | |  | |  | |  |  |  |  |  |  |
|  | |  | |  | |  |  |  |  |  |  |
| **3 nM VTX** | | **0 nM Cpd9** | | **125 nM Cpd9** | | **250 nM Cpd9** |  | **125 nM Cpd9** | **0 nM VTX** | **3 nM VTX** | **6 nM VTX** |
| **500 nM Cpd9** | | 0.000 | | 0.000 | | 0.003 |  | **12 nM VTX** | 0.000 | 0.000 | 0.910 |
| **250 nM Cpd9** | | 0.000 | | 0.001 | |  |  | **6 nM VTX** | 0.000 | 0.000 |  |
| **125 nM Cpd9** | | 0.000 | |  | |  |  | **3 nM VTX** | 0.000 |  |  |
|  | |  | |  | |  |  |  |  |  |  |
|  | |  | |  | |  |  |  |  |  |  |
| **6 nM VTX** | | **0 nM Cpd9** | | **125 nM Cpd9** | | **250 nM Cpd9** |  | **250 nM Cpd9** | **0 nM VTX** | **3 nM VTX** | **6 nM VTX** |
| **500 nM Cpd9** | | 0.000 | | 0.045 | | 1.000 |  | **12 nM VTX** | 0.000 | 0.000 | 1.000 |
| **250 nM Cpd9** | | 0.000 | | 0.303 | |  |  | **6 nM VTX** | 0.000 | 0.001 |  |
| **125 nM Cpd9** | | 0.000 | |  | |  |  | **3 nM VTX** | 0.000 |  |  |
|  | |  | |  | |  |  |  |  |  |  |
|  |  | |  | |  |  |  |  |  |  |  |
| **12 nM VTX** | | **0 nM Cpd9** | | **125 nM Cpd9** | | **250 nM Cpd9** |  | **500 nM Cpd9** | **0 nM VTX** | **3 nM VTX** | **6 nM VTX** |
| **500 nM Cpd9** | | 0.000 | | 0.926 | | 1.000 |  | **12 nM VTX** | 0.000 | 1.000 | 1.000 |
| **250 nM Cpd9** | | 0.000 | | 0.992 | |  |  | **6 nM VTX** | 0.000 | 0.999 |  |
| **125 nM Cpd9** | | 0.000 | |  | |  |  | **3 nM VTX** | 0.000 |  |  |
|  |  | |  | |  |  |  |  |  |  |  |

**Supplementary Table 6:** Statistics from viability of MV4-11 cells treated with Cpd **9** and VTX for 24 hours. P-values were calculated using one-way ANOVA with Tukey’s HSD post-hoc test. Significant values ≤0.05 are highlighted with red numbers. N=6

| **0 nM VTX** | **0 µM Cpd 9** | **0.25 µM Cpd 9** | **0.5 µM Cpd 9** |  | **0 µM Cpd9** | **0 nM VTX** | **3 nM VTX** | **6 nM VTX** |
| --- | --- | --- | --- | --- | --- | --- | --- | --- |
| **1.0 µM Cpd9** | 0.086 | 0.001 | 0.028 |  | **12 nM VTX** | 0.000 | 0.000 | 0.000 |
| **0.5 µM Cpd9** | 1.000 | 1.000 |  |  | **6 nM VTX** | 0.000 | 1.000 |  |
| **0.25 µM Cpd9** | 0.991 |  |  |  | **3 nM VTX** | 0.000 |  |  |
|  |  |  |  |  |  |  |  |  |
|  |  |  |  |  |  |  |  |  |
| **3 nM VTX** | **0 µM IM5** | **0.25 µM Cpd 9** | **0.5 µM Cpd 9** |  | **0.25 µM Cpd9** | **0 nM VTX** | **3 nM VTX** | **6 nM VTX** |
| **1.0 µM Cpd9** | 0.000 | 0.000 | 0.029 |  | **12 nM VTX** | 0.000 | 0.000 | 0.999 |
| **0.5 µM Cpd9** | 0.995 | 0.041 |  |  | **6 nM VTX** | 0.000 | 0.000 |  |
| **0.25 µM Cpd9** | 0.608 |  |  |  | **3 nM VTX** | 0.004 |  |  |
|  |  |  |  |  |  |  |  |  |
|  |  |  |  |  |  |  |  |  |
| **6 nM VTX** | **0 µM Cpd 9** | **0.25 µM Cpd 9** | **0.5 µM Cpd 9** |  | **0.5 µM Cpd9** | **0 nM VTX** | **3 nM VTX** | **6 nM VTX** |
| **1.0 µM Cpd9** | 0.000 | 0.003 | 0.213 |  | **12 nM VTX** | 0.000 | 0.000 | 0.286 |
| **0.5 µM Cpd9** | 0.000 | 0.986 |  |  | **6 nM VTX** | 0.000 | 0.030 |  |
| **0.25 µM Cpd9** | 0.026 |  |  |  | **3 nM VTX** | 0.000 |  |  |
|  |  |  |  |  |  |  |  |  |
|  |  |  |  |  |  |  |  |  |
| **12 nM VTX** | **0 µM Cpd 9** | **0.25 µM Cpd 9** | **0.5 µM Cpd 9** |  | **1.0 µM Cpd9** | **0 nM VTX** | **3 nM VTX** | **6 nM VTX** |
| **1.0 µM Cpd9** | 0.008 | 0.002 | 0.991 |  | **12 nM VTX** | 0.000 | 0.007 | 0.997 |
| **0.5 µM Cpd9** | 0.304 | 0.141 |  |  | **6 nM VTX** | 0.000 | 0.221 |  |
| **0.25 µM Cpd9** | 1.000 |  |  |  | **3 nM VTX** | 0.000 |  |  |

**Supplementary Table 7:** Statistics from viability of OCI-AML3 cells treated with Cpd **9** and VTX for 24 hours. P-values were calculated using one-way ANOVA with Tukey’s HSD post-hoc test. Significant p-values ≤0.05 are highlighted with red numbers. N=6.

| **0 µM VTX** | **0 µM Cpd9** | **0.25 µM Cpd9** | **0.5 µM Cpd9** | **1.0 µM Cpd9** |  | **0 µM Cpd9** | **0 µM VTX** | **2 µM VTX** | **4 µM VTX** |
| --- | --- | --- | --- | --- | --- | --- | --- | --- | --- |
| **2.0 µM Cpd9** | 0.002 | 0.005 | 0.620 | 0.875 |  | **6 µM VTX** | 0.000 | 0.000 | 0.003 |
| **1.0 µM Cpd9** | 0.535 | 0,727 | 1.000 |  |  | **4 µM VTX** | 0.050 | 1.000 |  |
| **0.5 µM Cpd9** | 0.817 | 0,933 |  |  |  | **2 µM VTX** | 0.638 |  |  |
| **0.25 µM Cpd9** | 1.000 |  |  |  |  |  |  |  |  |
|  |  |  |  |  |  |  |  |  |  |
| **2 µM VTX** | **0 µM Cpd9** | **0.25 µM Cpd9** | **0.5 µM Cpd9** | **1.0 µM Cpd9** |  | **0.25 µM Cpd9** | **0 µM VTX** | **2 µM VTX** | **4 µM VTX** |
| **2.0 µM Cpd9** | 0.000 | 0.000 | 0.000 | 0.000 |  | **6 µM VTX** | 0.000 | 0.002 | 0.019 |
| **1.0 µM Cpd9** | 1.000 | 1.000 | 1.000 |  |  | **4 µM VTX** | 0.358 | 1.000 |  |
| **0.5 µM Cpd9** | 0.999 | 0.999 |  |  |  | **2 µM VTX** | 0.804 |  |  |
| **0.25 µM Cpd9** | 1.000 |  |  |  |  |  |  |  |  |
|  |  |  |  |  |  |  |  |  |  |
| **4 µM VTX** | **0 µM Cpd9** | **0.25 µM Cpd9** | **0.5 µM Cpd9** | **1.0 µM Cpd9** |  | **0.5 µM Cpd9** | **0 µM VTX** | **2 µM VTX** | **4 µM VTX** |
| **2.0 µM Cpd9** | 0.000 | 0.000 | 0.000 | 0.000 |  | **6 µM VTX** | 0.000 | 0.072 | 0.009 |
| **1.0 µM Cpd9** | 1.000 | 0.977 | 0.971 |  |  | **4 µM VTX** | 1.000 | 1.000 |  |
| **0.5 µM Cpd9** | 1.000 | 1.000 |  |  |  | **2 µM VTX** | 0.991 |  |  |
| **0.25 µM Cpd9** | 1.000 |  |  |  |  |  |  |  |  |
|  |  |  |  |  |  |  |  |  |  |
| **6 µM VTX** | **0 µM Cpd9** | **0.25 µM Cpd9** | **0.5 µM Cpd9** | **1.0 µM Cpd9** |  | **1.0 µM Cpd9** | **0 µM VTX** | **2 µM VTX** | **4 µM VTX** |
| **2.0 µM Cpd9** | 0.000 | 0.000 | 0,000 | 0,000 |  | **6 µM VTX** | 0.000 | 0.000 | 0.056 |
| **1.0 µM Cpd9** | 1.000 | 0.999 | 1,000 |  |  | **4 µM VTX** | 0.790 | 0.754 |  |
| **0.5 µM Cpd9** | 1.000 | 1.000 |  |  |  | **2 µM VTX** | 1.000 |  |  |
| **0.25 µM Cpd9** | 1.000 |  |  |  |  |  |  |  |  |
|  |  |  |  |  |  |  |  |  |  |
|  |  |  |  |  |  | **2.0 µM Cpd9** | **0 µM VTX** | **2 µM VTX** | **4 µM VTX** |
|  |  |  |  |  |  | **6 µM VTX** | 0.000 | 0.002 | 0.628 |
|  |  |  |  |  |  | **4 µM VTX** | 0.000 | 0.807 |  |
|  |  |  |  |  |  | **2 µM VTX** | 0.000 |  |  |
